# Microplastics in the Asia-Pacific Region in the Plasticene Era: Exposures and Health Risks

**DOI:** 10.5334/aogh.4326

**Published:** 2024-01-31

**Authors:** Peter Sly, Khadija Al Nabhani, Kam Sripada, Fujio Kayama

**Affiliations:** 1 Children’s Health and Environment Program, Child Health Research Centre, The University of Queensland, AU; 2 University of New South Wales, AU; 3 Centre for Digital Life Norway, Institute of Biotechnology & Food Science, Norwegian University of Science & Technology, NO; 4 Division of Environmental Medicine, Center for Community Medicine, Jichi Medical University, JP

**Keywords:** Plastic products, human health, environmental exposure, environmental degradation, the Pacific Basin Consortium for Environment and Health

## Abstract

Within the broader Anthropocene Epoch resides the Plasticene Era, where humans are subjected pervasively to nano- and microplastics (NMPs). Human’s widespread exposure with NMPs occurs through the air we breathe, water we drink, and food we eat. NMP sources are wide and varied; atmospheric NMPs are largely attributed to fibres from car tyres and synthetic clothing, while particles from food packaging, personal care products, and plastic manufacturing contribute significantly to food and water contamination. NMPs have become inherent within the human body and have been found in every organ. As such, the evidence base around adverse health effects is fragmented but growing. This article presents a mini-review and report of sessions presented about NMPs at the 19th International Conference of the Pacific Basin Consortium for Environment and Health, held on Jeju Island, in 2022. Abundant evidence of substantial exposure to NMPs in the Asia-Pacific region has been exhibited. Addressing this issue necessitates the collaboration of policymakers, manufacturers, and researchers to develop safer alternatives and implement mitigation and remediation strategies. The ongoing development of a new United Nations-led global plastic treaty presents a crucial opportunity that must be acted on and not be compromised.

This article presents a mini-review and report of sessions on NMPs presented at the 19th International Conference of the Pacific Basin Consortium for Environment and Health, held on Jeju Island, in 2022.

## The Plasticene Era

Plastics, from the Greek word *plastikos*, meaning ‘capable of being molded’ [1], encompass a wide range of synthetic or semi-synthetic materials composed of long chains of polymers. While the first known plastic, Bakelite, was invented in 1907 [2], widespread production of plastics did not begin until the 1950s, accelerating in the early 2000s. According to a recent report from the United Nations Environment Program, plastic production exceeds 400 million tonnes, with only 12% incinerated, 9% recycled, with the rest entering landfills or in the environment, including oceans [3].

Plastics are transforming our world with great influence, as geological research reveals plastics evident in earth cores, rivers and lakes, and ocean sediments, even in secluded areas far from human habitation [4,5,6,7,8,9,10]. As the likelihood that the plastic components of our environment are permanent with the potential to exist in future fossils this has led to the naming of the current era as the Plasticene Era – a subset of the Anthropocene Era [11,12,13,14]. Consequently, new lexicon is emerging to describe the characteristics of this new era (**Box 1**).

**Box 1 A Plasticene Lexicon.^a^**

^a^Adapted from Haram *et al*. in *The Marine Pollution Bulletin* and Minogue in *Shorelines* [11,12] **Microplastics in the environment**.

***Plasticene:*** The Age of Plastics, a new age in Earth’s history that began with the proliferation of plastic and plastic products in the 1950s.

***Plastisphere:*** Communities of organisms, including microorganisms, bivalve molluscs, fish, etc., colonizing plastics.

***Epiplastics:*** organisms living on the surface of plastics. This originally referred to microorganisms on floating plastics under experimental conditions

***Plasticized:*** organisms, animals or environments made “more plastic” by plastic pollution.

***Plastivore:*** organisms that eat plastic, primarily inadvertently, mistaking plastics for food.

***Plastic cycle:*** the complex movement of plastics through ecosystem compartments.

***Plastiglomerate:*** a dense material formed by molten plastics fusing with hard materials, such as rocks.

***Plasticrust:*** crusts made of plastics attached to tropical or sub-tropical rocks and rock surfaces.

***Pyroplastic:*** a fused mass of melted plastics not containing other materials and light enough to float in seawater.

***Plastictrash:*** any type of garbage made of plastics.

***Plastic Confetti:*** small plastic fragments formed by the degradation of larger plastics.

***Nurdle:*** plastic pellet used in the manufacture of plastic products, found on beaches worldwide.

Degradation of plastics generally does not occur through natural processes such as biodegradation, but through physical and chemical processes which decompose into nano- or microplastics (NMPs) [15,16]. NMPs can take the form of particles or fibres, with varying sizes and lengths [5,17] (**Box 2**). Among the classified plastics listed in **Box 2**, microplastics have been found in the ocean, fresh waters, sediments, soils, and in the air [18]. Environmental NMPs have been found around human settlements, especially coastal communities, but also in remote locations including polar regions and deep ocean troughs [18]. NMPs contaminate drinking water [19] and food via contaminated soils [20], contaminated aquatic ecosystems [21,22], and through food packaging [23]. Waste mismanagement and transport of plastics debris are considered a source of NMPs due to maritime activities, recreational fishing, and debris thrown by cruise or private ships between remote islands [24]. Wear and tear of car tyres and fibres from synthetic textiles are considered major sources of NMP contamination [5].

**Box 2 Classification of plastics.^a^**

^a^Adapted from Hartmann *et al*. in *Environmental Science and Technology* [24].

**Macroplastics**: plastics > 1 cm in at least one dimension

**Mesoplastics:** plastics 1 to <10 mm

**Microplastics**: plastics with dimensions between 1 and <1000 µm

- Primary microplastics: originally made to be micronized, usually for cosmetics
- Secondary microplastics: broken down from macroplastics in the environment

**Nanoplastics**: plastics 1 to <1000 nm

Several plastic cycles facilitate the movement of NMPs from various sources to humans. In attempts to decrease the prevalence of microplastics, direct manufacture of these plastics for personal use – such as microbeads – has been restricted. However, NMPs are still predominant in society due to the decomposition of larger plastics into secondary NMPs. Food contact materials and containers shed NMPs directly onto food consumed by humans, including plastic infant feeding bottles [25,26]. Plastic manufacturing, plastic recycling activities, and breakdown of plastics in landfill, resulting in contamination of surface or ground water sources. NMPs from car tyres can be easily tracked into homes and contaminate household dust, which is commonly ingested by children through hand-to-mouth behaviour. NMPs in contaminated water enter the aquatic food chain, accumulating in fish that are consumed by humans [21,22]. Further examples include NMPs in oceans being effectively aerosolised and taken up into clouds, which are transported over landmasses and rain down on land, contaminating soils and vegetation in the process [27]. NMPs in contaminated vegetation are eaten by animals, enter the human food chain and thus accumulate in humans [18]. Due to the hydrophobic nature of plastics, they carry persistent organic pollutants such as heavy metals and toxic chemicals [62]. Therefore, NMPs can impact humans in sites separate from where they originated from.

## Exposure Routes for Microplastics

Microplastics in the environment result in human exposure through multiple routes including, ingestion, inhalation, trans-dermal transfer, trans-placental exposures, and in breast milk [5,17,18,19,21,28,29,30,31,32]. Inhalation and ingestion are the likely major exposure routes in children and adults, while trans-placental transfer, exposure in breast milk, and non-nutritive ingestion of NMPs in house dust are main exposure routes in early development of children [29,32].

## Health Effects of Microplastics

The effects of NMPs on human health are eliciting research attention but remains understudied. Microplastics can be found in various human organs and body secretions including the brain, lungs, heart, ovaries, uterus, placenta, testes, gastrointestinal tract, liver, spleen, blood, sputum, semen, breastmilk, meconium, and faeces [17,33,34,35,36,37,38,39,40,41,42,43,44,45]. Exposure to NMPs have been correlated with immune dysregulation, increased intestinal permeability and alterations in gastrointestinal microbiome, cancer, neurotoxicity, oxidative stress and inflammation, infertility, endocrine disruption, developmental toxicity and genotoxicity in animals and humans [14,15,18,31,36,37,40,46,47,48].

Adverse health effects can arise from direct physical interaction between NMPs and human cells and tissues [17,18,23,29,30,31,49] and from chemical interactions with NMP components [14,46,48]. Direct physical effects have been less well-studied, but the available reports state a possible relationship between microplastics from school floors with students’ eye and lower airway irritation [50], the potential of NMPs to deposit and cause inflammatory reactions in sensitive tissues e.g., alveoli with limited removal processes [33,51], as well as health effects from occupational exposures in plastic industry workers [52]. NMPs contain plasticisers and other additives and are known to adsorb environmental chemicals with known toxicity [13]. A recent review of this area highlighted the possible connection between the use of chemical additives and dyes, including phthalates, bisphenols, styrene, and polybrominated diphenyl ethers to reproductive and developmental toxicity and allergies. In addition, secondary toxins, including heavy metals and persistent organic pollutants, may be correlated with immunotoxicity. Furthermore, direct physical interactions has possible links to respiratory disease, inflammation, oxidative stress, cytotoxicity, and lipid accumulation [18]. Moreover, the hydrophobic surface of NMPs is conducive for microbial colonisation and biofilm formation. Therefore, instances of human exposure to NMPs accompanied with microorganisms have been associated with infection, antimicrobial resistance (by the dissemination of antibiotic resistance genes), and gut dysbiosis [18,53]. Children exhibit different patterns of exposures and vulnerabilities to NMPs, as also evidenced with other environmental toxicants [33]. Nevertheless, further research is necessary to elucidate the true impact of NMP exposure on human health, including defining the windows of susceptibility for various adverse health outcomes.

## Microplastic Exposure in the Asia-Pacific Region

NMP research is expanding within the Asia-Pacific region, with an increasing number of studies examining environmental distribution within the region. In South Korea, expanded polystyrene (EPS, commonly known as Styrofoam) debris is common in the marine environment, originating mainly from fishing and aquaculture buoys [54]. EPS contains hexabromocyclododecane and is common along the Asia-Pacific coastal region. Suspected tsunami debris containing EDS is thought to originate from Alaskan beaches and demonstrates the potential for long range ocean transport of EDS [54]. Research has also demonstrated the possibility of NMP fibres to travel via sea air, depositing in oceans and terrestrial rivers in the Asia-Pacific region [55]. A study examining the horizontal and vertical distribution of NMPs in Korean coastal waters showed that, while NMP abundance was greatest in surface water, NMPs were present through the water column to depths of 58 m [56]. Notably, NMP abundance was significantly greater near urban areas than in rural areas. In addition, the presence of low density NMPs was in higher concentrations at deeper depths than what has been approximated from natural physical processes, such as the movement of the water [56]. Several studies have examined NMPs in Japanese small-scale river sediments [7,8,57], indicating the ubiquitous nature of NMPs, albeit with relatively modest concentrations compared to other rivers around the world. A study dating sediment cores from Tokyo Bay suggested that NMP pollution emerged in the 1950s, intensifying in the 2000s [9]. Similar trends were observed in sediment cores collected in Thailand, Malaysia, and South Africa [9]. In these regions, NMPs are also common in surface road dust, primarily derived from food packaging and tyres [58]. In Australia, NMPs are also found in river sediment and ocean environments, following similar patterns to those reported globally, with factors such as urbanisation, human coastal population size, and tidal river flow dynamics correlating with NMP adundance [6,59,60,61,62]. NMPs were found in blue mussels (*Mytilus spp*.) [60] from South Australian waters previously thought to be pristine, and in fresh water shrimp (*Parataya australiensis*) in Victorian rivers [61]. NMP fibres, specifically polyester and rayon, are common in Australian rivers [61]. Sediments collected from stormwater drains in Perth, within the State of Western Australia, were identified as a path for NMPs to enter the marine environment [63]. Polyethylene and polypropylene were predominant NMP fibres in this study [63].

## Microplastic Presentations at the Pacific Basin Consortium for Environment and Health

Microplastic research was featured prominently at the 19^th^ International Conference of the Pacific Basin Consortium of Environment and Health, held recently in Jeju, South Korea [https://pacificbasin.org/conferences-2/2022-international-meeting/]. Two plenary sessions were dedicated to NMPs. One session titled “Health effects of plastics exposure across the life span” was hosted by the Minderoo Foundation [https://www.minderoo.org/], highlighting how humans are exposed to NMPs along with theiradverse health effects. Two presentations highlighted the scope of the problems and the vast array of adverse effects on human health. Another presentation highlighted the deficiencies of the regulatory framework and how this leads to a lack of protection for the global population. Also within the Minderoo session was an enlightening presentation focused on pathways to safer plastics. Taken together, the presentations in the Minderoo session posit the necessity to recognise that the return to a plastic-free world is not possible, and thus the way forward is to develop plastics that do not harm the environment or the health of the population. Methods discussed included the use of biopolymers with similar mechanical properties as conventional plastic polymers produced from natural resources: such as plant oils, sugars or cellulose biomass to make bioplastics; advanced recycling technologies like depolymerization whereby plastic is broken in monomers, or solvolysis in which plastics are dissolved, purified and the polymer chains reprecipitated to reconstitute the plastic; or substitution of harmful additives with safer alternatives. Since the conference, the Minderoo Foundation have collaborated in producing a very comprehensive review of ‘all things plastic’ [64]. Interested readers are advised to consult this excellent publication for further information.

The other session presented primary research conducted within the region. Findings presented included: the effects of NMPs in Korean ecosystems and their implications for human health; a demonstration that healthy pregnant women in Indonesia all had multiple NMPs in their stool, showing the ubiquitous nature of exposure; and the levels of NMP exposure in coastal areas in Malaysia and Fiji.

## Summary

We certainly live in a “plastic world”, but unlike the lyrics from the Aqua classic, “life in plastic” is not “fantastic”. Exposure to, and problems from NMPs are escalating, and a global solution is urgently required. Plastics producers should take proactive measures to prevent the release of NMPs through thoughtful design and must demonstrate that products are safe for children before bringing them to market. Even with the potential development of safer plastics, the population is still confronted with seven decades of NMP exposure. As such, the ongoing development of a strong global plastic treaty at the United Nations is an opportunity that must not be compromised.
